# Acute pancreatitis followed by retroperitoneal perforation of the descending colon and a duodenal fistula: Report of a case

**DOI:** 10.1016/j.ijscr.2020.05.095

**Published:** 2020-06-11

**Authors:** Kentaro Yoshikawa, Alan Kawarai Lefor, Tadao Kubota

**Affiliations:** a Department of Surgery, Tokyo Bay Urayasu Ichikawa Medical Center, 3-4-32 Toudaijima, Urayasu, Chiba 279-0001, Japan; b Department of Surgery, Jichi Medical University, 3311-1, Yakushiji, Shimotsuke, Tochigi 329-0498, Japan

**Keywords:** Acute pancreatitis, Colon perforation, Retroperitoneal drainage, Loop ileostomy

## Abstract

**Introduction:**

There are several reports of colon perforation in patients with acute pancreatitis, but the mechanism is not understood. We describe a patient with acute pancreatitis followed by retroperitoneal perforation of the descending colon and a duodenal fistula.

**Case presentation:**

A 51-year-old male presented with acute pancreatitis. He was hemodynamically unstable, had respiratory failure on admission and was treated in the intensive care unit (ICU). He recovered and left the ICU on day 13. Although his general condition improved, a computed tomography (CT) scan showed air and fluid in the left retroperitoneum. Gastrografin enema and CT scan showed extraluminal leakage in the descending colon and retroperitoneal drainage was performed on day 27. After drainage, there was continuous voluminous feculent discharge, and a loop ileostomy was performed on day 34. A repeat CT scan revealed ascites. A percutaneous catheter injected with contrast showed a duodenal fistula. After drainage, the fever resolved and the patient was discharged on hospital day 106.

**Discussion:**

Although there is no clear mechanism of colonic perforation in patients with acute pancreatitis, one hypothesis is that ischemia secondary to inflammation caused by pancreatitis plays a role. The involved area is usually in the watershed areas of the colon. Retroperitoneal drainage of the colon perforation may have necessitated creation of a diverting loop ileostomy.

**Conclusion:**

Retroperitoneal colon perforation must be considered in patients with acute pancreatitis.

## Highlights

• Retroperitoneal perforation of the colon must be considered in patients with acute pancreatitis.
• The most common site of perforation is the transverse and descending colon.
• Retroperitoneal drainage may lead to expansion of the perforation site, necessitating a diverting stoma.

## 1 Introduction

There are several reports of colon perforation in patients with acute pancreatitis, but the mechanism is not fully understood. We describe a patient with acute pancreatitis followed by retroperitoneal perforation of the descending colon. This work is reported in conformity with the Surgical CAse REport (SCARE) Guidelines [1].

## 2 Case presentation

A 51-year-old male presented with epigastric pain and was diagnosed with acute pancreatitis at an outside facility. Despite fluid resuscitation, there was no improvement and he was transferred to our hospital the following day. His medical history was remarkable for hypertension and hyperuricemia. He drinks alcohol (56 g) every day. Physical examination revealed temperature 36.4 °C, blood pressure 138/101 mmHg, pulse 121/min, and respiratory rate 22/min. He was in acute distress with tenderness in the epigastrium without rebound tenderness. Laboratory data included white blood cell (WBC) count of 11,900/μl, serum creatinine 2.28 mg/dl, total bilirubin 1.14 mg/dl and C-reactive protein 32 mg/dl. Serum lipase was 894 U/L. Computed tomography scan showed peripancreatic and a mesocolic fluid collection classified as Balthazar grade E.

**Fig. 1:**
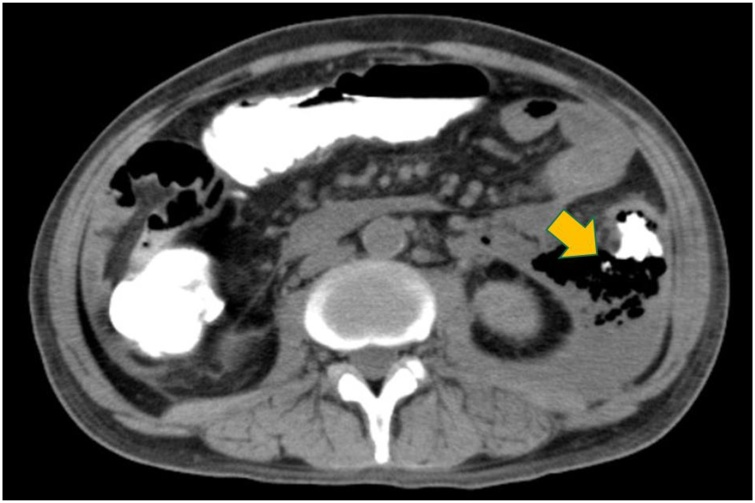
Extravasation of contrast material from the descending colon (arrow).

On the day of admission, aggressive fluid resuscitation continued for the treatment of pancreatitis. With continued tachycardia and respiratory failure (SpO2 88% mask 5 L), he was brought to the intensive care unit (ICU) and supported with mechanical ventilation. By hospital day 5, there was improvement in the respiratory failure and renal failure. He was extubated and left the ICU to a general ward on hospital day 13. Although the patient appeared to be in no distress, the fever and elevated WBC continued after leaving the ICU. On hospital day 23 a CT scan was obtained which showed a gas and fluid collection in the left retroperitoneum. There was no contrast in one segment of the wall of the descending colon and perforation was suspected. To confirm the perforation, a contrast enema was performed after the CT scan which showed extraluminal contrast adjacent to the descending colon (Fig. 1). This conformed the diagnosis of a perforation of the descending colon.

**Fig. 2:**
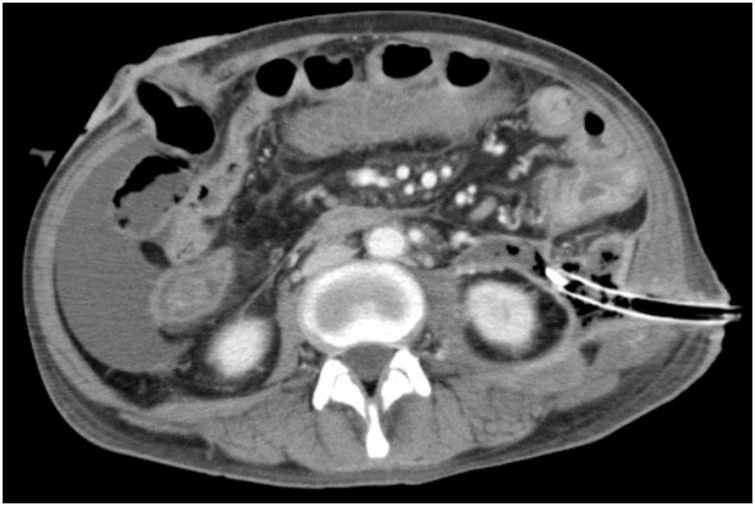
Retroperitoneal drainage through the left flank revealed a large amount of feculent discharge, necessitating a diverting loop ileostomy.

**Fig. 3:**
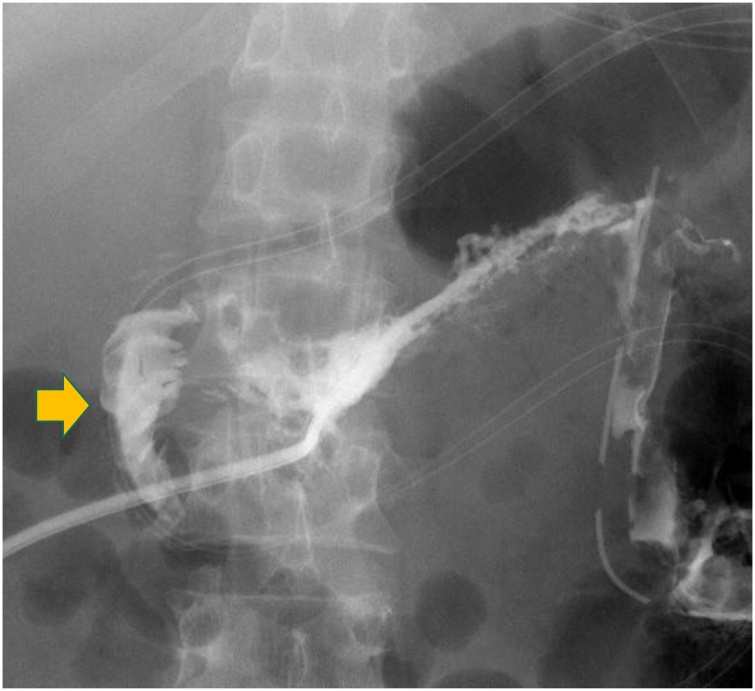
Duodenal fistula was demonstrated with injection of radiopaque contrast material through a percutaneous catheter (arrow).

To control leakage from the perforation, retroperitoneal drainage was established by placing a 28Fr catheter percutaneously. As the drain was inserted, feculent material spontaneously drained, with a total of 700 mL. To control the leak, a loop ileostomy was performed for diversion (Fig. 2) on hospital day 34. After the diversion, discharge from the retroperitoneal drain decreased but fever persisted. A CT scan at that time revealed ascites. A 12Fr catheter was placed through the abdominal wall and water soluble contrast injected which demonstrated a duodenal fistula (Fig. 3). The fever resolved after drainage of the ascites. Previously placed drains in the retroperitoneum were removed after the volume of discharge decreased and the patient was discharged on hospital day 106.

## 3 Discussion

There are several reports of colon perforation in patients with acute　pancreatitis. An extensive search was conducted (http://www.pubmed.com) for previous studies related to this topic, using the following search terms: “pancreatitis” and “colon perforation.” A total of 19 previously reported patients were identified and are summarized in Table 1.

**Table 1:** Previously reported patients with colon perforation associated with acute pancreatitis.

No.	Author	Year	Country	Age	Gender	Cause of pancreatitis	Perforation site	Days to surgery	Treatment	Outcome
1	Dhadlie [12]	2019	Australia	72	M	Unknown	Ascending colon	7	Right-sided hemicolectomy with end ileostomy	Alive
2	Hozaka [6]	2018	Japan	31	M	Gallstone	Descending colon	36	Ileostomy + Video-assisted retroperitoneal debridement	Alive
3	Nakanishi [3]	2015	Japan	72	M	Alcohol	Descending colon	15	Colectomy	Alive
4				38	M	Alcohol	Ascending colon	6	Colectomy	Alive
5				78	M	Alcohol	Transverse colon	11	Colectomy	Alive
6				59	M	ERCP	Ascending colon	47	Colectomy	Alive
7	Nagpal [11]	2015	India	54	M	Gallstone	Transverse colon	32	Sub-total colectomy + Ileostomy	Dead
8				17	M	Idiopathic	Transverse colon	84	Sub-total colectomy + Ileostomy	Alive
9				52	M	Trauma	Transverse colon	14	Sub-total colectomy + Ileostomy	Alive
10				75	F	Idiopathic	Hepatic flexure	26	Sub-total colectomy + Ileostomy	Alive
11				35	M	Gallstone	Splenic flexure	30	Sub-total colectomy + Ileostomy	Alive
12	Gondal [9]	2014	USA	59	M	Gallstone	Descending colon	11	Repair of the perforation + Drains + Jejunostomy	Alive
13	Pauli [4]	2013	USA	67	M	Gallstone	Descending colon		Fully covered over-the-scope(OTS) stent (later, segmental colectomy)	Alive
14	Aghenta [7]	2009	USA	71	M	Alcohol	Splenic flexure		CT guided percutaneous drainage	Alive
15	Han [10]	2008	Taiwan	83	F	Gallstone	Transverse colon	19	Necrosectomy + Diverting loop ileostomy + Drainage of the abscess	Alive
16	Familiari [5]	2002	Italy	73	M		Transverse colon		Endoscopic clip	Alive
17	Jover [14]	1996	Spain	28	M	Alcohol			Percutaneous drainage	Alive
18	Cho [8]	1996	Korea	63	M		Splenic flexure	57	Colectomy	Alive
19	Fazio [13]	1973	USA	38	M	Alcohol	Transverse colon	10	Loop transverse colostomy	Alive

**Fig. 4:**
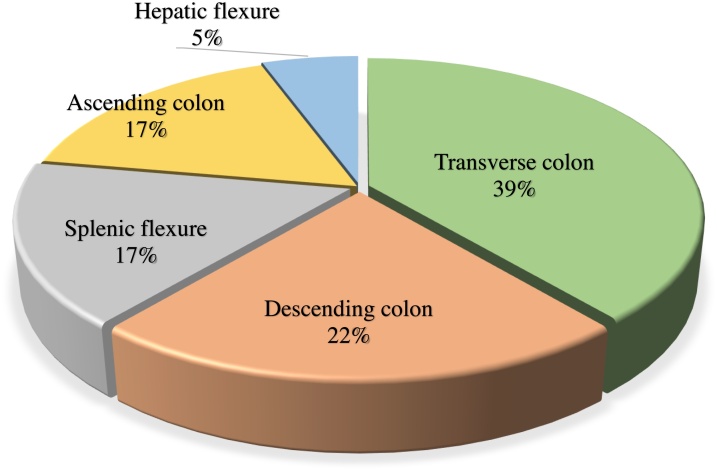
Site of perforation in previously reported patients with colon perforation associated with pancreatitis.

Although the mechanism of colon perforation in patients with acute pancreatitis is not fully understood, there are several theories. One is that direct retroperitoneal spread of pancreatic enzymes to the mesocolon, causes pericolitis, transmural necrosis, and perforation [2]. A fluid collection in at least 2 locations (Balthazar grade E) on the initial CT scan is considered a risk factors for colon perforation [3]. From the data in Table 1, the rate of perforation is greatest in the transverse colon and descending colon (Fig. 4). This may be due to the fact that the splenic flexure is a vascular watershed area prone to poor perfusion, which may ultimately cause ischemia.

In this patient, the main site of inflammation was pancreatic body and tail, which led to perforation of the duodenum and descending colon. After diagnosing the colon perforation by contrast enema and CT scan, we chose to perform retroperitoneal drainage, which may have applied pressure at the perforation site and leading it to expand with persistent feculent drainage. The decision to perform retroperitoneal drainage near the site of colon perforation resulted the need for a in loop ileostomy. From the data in Table 1, most authors describe performing a colectomy and ileostomy similar to treatment of the present patient, but there are also reports of closing the perforation site with an endoscopic clip or enteral stent followed by planned colon resection [4,5]. Using these devices prior to the retroperitoneal drainage might have prevented the need for a loop ileostomy.

## 4 Conclusion

Retroperitoneal colon perforation must be considered in patients with acute pancreatitis. If the patient is clinically stable, closing the perforation site using an endoscopic clip or enteral stent followed by planned colon resection is a reasonable treatment option which may avoid the need for an ileostomy.

## Funding

This research did not receive any specific grant from funding agencies in the public, commercial, or not-for-profit sectors.

## Ethical approval

Ethical approval was exempted by our institution.

## Consent

Written informed consent was obtained from the patient for the publication of this case report and accompanying images. A copy of the written consent is available and can be reproduced whenever needed.

## Author contribution

Kentaro Yoshikawa gathered patient’s date, designed the case report, and drafted manuscript. Tadao Kubota and Alan Kawarai Lefor supervised the report. All authors read and approved the final manuscript.

## Registration of research studies

This work is case report and there is no need of registration.

## Guarantor

Dr. Kentaro Yoshikawa.

## Provenance and peer review

Not commissioned, externally peer-reviewed.

## Declaration of Competing Interest

None of the authors has any conflict of interest to declare.
